# Investigation of Sm Addition on Microstructural and Optical Properties of CoFe Thin Films

**DOI:** 10.3390/ma16155380

**Published:** 2023-07-31

**Authors:** Wen-Jen Liu, Yung-Huang Chang, Chia-Chin Chiang, Jian-Xin Lai, Yuan-Tsung Chen, Hsiung-Liang Chen, Shih-Hung Lin

**Affiliations:** 1 Department of Materials Science and Engineering, I-Shou University, Kaohsiung 840, Taiwan; jurgen@isu.edu.tw; 2 Bachelor Program in Industrial Technology, National Yunlin University of Science and Technology, 123 University Road, Section 3, Douliou, Yunlin 640301, Taiwan; changyhu@yuntech.edu.tw; 3 Department of Mechanical Engineering, National Kaohsiung University of Science and Technology, Kaohsiung City 80778, Taiwan; ccchiang@nkust.edu.tw; 4 Graduate School of Materials Science, National Yunlin University of Science and Technology, 123 University Road, Section 3, Douliou, Yunlin 640301, Taiwan; jianxin19971124@hotmail.com (J.-X.L.); cody0975760755@gmail.com (H.-L.C.); 5 Department of Electronic Engineering, National Yunlin University of Science and Technology, 123 University Road, Section 3, Douliou, Yunlin 640301, Taiwan; isshokenmei@yuntech.edu.tw

**Keywords:** Co_40_Fe_40_Sm_20_ films, sputtering, annealing, surface roughness, nanomechanical properties, optical transparency

## Abstract

CoFe-based alloys and rare earth (RE) elements are among the most studied materials in applying magnetic devices to improve soft magnetic characteristics. A series of Co_40_Fe_40_Sm_20_ films are deposited on a glass substrate via the sputtering technique, followed by an annealing process to investigate their effect on microstructural and optical properties of Co_40_Fe_40_Sm_20_ films. In this study, the increase in the thickness of Co_40_Fe_40_Sm_20_ films and annealing temperatures resulted in a smoother surface morphology. The 40 nm Co_40_Fe_40_Sm_20_ films annealed 300 °C are expected to have good wear resistance and adhesive properties due to their high values of H/E ratio and surface energy. Optical transparency also increased due to the smoother surface of the Co_40_Fe_40_Sm_20_ films.

## 1. Introduction

Magnetism and nanostructured magnetic devices have been recently developed because of their high potential in the application of magnetic sensors, spintronics, and magnetic storage technologies. Cobalt-iron (CoFe) alloys are essential soft magnetic materials with high saturation magnetization (M_s_), high Curie temperature (T_c_), high permeability, good thermal stability, and giant magnetostriction (λ_s_). However, these alloys are extremely brittle and do not possess low coercivity (H_c_). Thus, adding alloying elements is the primary way to reduce their H_c_ value and improve their mechanical properties to obtain optimum soft magnetic properties (low H_c_ and high M_s_) [1,2,3]. Research has shown that adding 1.7 to 2.1% vanadium (V) to CoFe alloys can provide a composition that possesses significantly improved strength and simultaneously retains satisfactory magnetic properties [4]. Rare-earth transition-metal (RM-TE) compounds are among the most favored candidates for magnetic and optical applications. 4f rare earth (RE) has more significant magnetic moments than 3d transition metals and may improve magnetic properties. Rakesh Kumar Singh et al. synthesized Ni_0.5_Zn_0.5_Fe_1.95_R_0.05_O_4_ nanoparticles (R = Pr, Sm, and La) via the citrated precursor method [5]. They reported that lattice constant and crystal size decreased after doping RE elements, thereby reducing coercivity, saturation magnetization, and magnetic exchange. In addition, dielectric loss was reduced from 100 Hz to 1 MHz, and samarium (Sm)-doped Nickel-Znic (NiZn) ferrite showed the smallest dielectric constant among all the samples. It obtained the partial substitution of cobalt (Co) ferrite with a small amount of Sm and gadolinium (Gd) via the citrate precursor route [6]. The research showed that substituting Sm or Gd enhanced crystal structure, microstructure, and magnetic properties. The RE elements presented a larger ionic radius, in which Sm ions (1.08 Å) were larger than Co (0.65 Å) and Fe (0.55 Å) ions when substituting cations with smaller ionic radii in other types of structures, can determine a change in cell symmetry and thus generate internal stress. Therefore, not only are the material’s structural properties changed, such as increased cell parameters, decreased grain dimensions, and average crystalline size, but also the dielectric, magnetic, and magnetostrictive properties of substituted materials [7]. RE metals with partially filled 4f and empty 5d orbitals have the potential to enhance photo-induced electron-hole pair separation [8]. Li et al. determined that SmCo with 37.7 at. % Sm has a good hard magnetic property with a high H_c_ of 37.938 kOe and M_s_ of 150 emu/cm^3^ [9]. Additionally, for application in high-density data storage, coercivity must be low enough to allow writing and overwriting, and it also must be large enough to provide long-term thermal stability [10]. Hence, Sm with an electronic configuration of 6s^2^4f^6^, which may provide a high magnetic moment, has been selected in this study as an additional alloying element [11]. Since the manufacturing and packaging processes during practical applications necessarily involve contact loading, the mechanical properties of magnetic thin films must be fully recognized, especially in the nano-scale regime. For this reason, the nanoindentation technique has been widely used to obtain mechanical characteristics, such as hardness, Young’s modulus, and elastic/plastic deformation behaviors of various thin films [12,13]. The optimum parameters of magnetic properties, such as coercivity, saturation magnetization, remanence magnetization, and magnetic anisotropy, are strongly affected by the crystallinity, surface roughness, and film thickness of ferromagnetic materials, which growth parameters and the annealing process can tune [1,14]. In this study, the CoFeSm films were fabricated on glass substrates, and the influence between microstructural and optical properties of CoFeSm films with various film thicknesses and annealing temperatures was examined. The significance of the work is investigating surface roughness in order to study the relationship between surface energy and magnetic–optical properties in CoFeSm thin films of various thicknesses and annealed at different temperatures. Moreover, the outcome of the present study is compared with the magnetic and adhesive characteristics of Cobalt Iron Vanadium (CoFeV), Cobalt Iron Tungsten (CoFeW), and Cobalt Iron Ytterbium (CoFeYb), and Cobalt Iron Yttrium (CoFeY) in Table 1 [15,16,17,18,19,20].

## 2. Materials and Methods

CoFeSm films were deposited onto a glass substrate with film thickness ranging from 10 nm to 50 nm at room temperature (RT) via direct-current (DC) magnetron sputtering, which employs an alloy target with 40 at. % Co, 40 at. % Fe, and 20 at % Sm. A glass substrate was chosen for this study due to its transparency in the near-infrared region (NIR)-visible region and low cost [8]. The investigations were carried out on as-deposited films and after various annealing treatments at 100 °C, 200 °C, and 300 °C, respectively, for 1 h in an Argon (Ar) environment. Before sputtering, glass substrates were pre-cleaned in acetone, ethanol, and deionized (DI) water for 10 min via ultrasonic cleaner and dried with N_2_ gas. The power density was 1.65 W/cm^2^, and the deposition rate was 1.2 nm/min. Before sputtering, the base pressure of the sputtering chamber was kept at 3.0 × 10^−7^ Torr. During deposition, the pressure was set at 3.5 × 10^−5^ Torr, the flow rate of argon gas was kept at 20 sccm, the power was set at 50 W, and the substrates were rotated at a speed of 20 rpm. After deposition, the samples were subjected to annealing at temperatures ranging from 100 °C to 300 °C for 60 min with a fixed heating rate of 50 °C/min. During the process of annealing, the pressure of the vacuum chamber was maintained at 2.7 × 10^−3^ Torr. CoFeSm films were cooled to RT via furnace cooling after the annealing process. The crystal structure of the CoFeSm films was identified using an X-ray diffractometer (XRD, PAN analytical X’pert PRO MRD, Davis, CA, USA). The surface roughness of the thin films was determined via atomic force microscopy (AFM, NanoMagnetics Instruments, ezAFM, Ulm, Germany). The contact angle of the CoFeSm films were evaluated via contact angle meter (CAM-110, Creating Nano Technologies, Tainan, Taiwan), and the surface energy of the films were calculated using the contact angle of water and glycerol on the thin films. The surface energy of Co_40_Fe_40_Sm_20_ films was calculated using the Owens–Wendt–Kaelble model, also known as the two-liquid model. In this model, the work of adhesion is shown in Equation (1) below [21]:

(1) $$\;W_{A}=2\left[\sqrt{\left(\gamma_{l}^{d}\right)\left(\gamma_{s}^{d}\right)}+\sqrt{\left(\gamma_{l}^{p}\right)\left(\gamma_{s}^{p}\right)}\right]$$

where $\gamma_{l}^{d}$ and $\gamma_{l}^{p}$ are the dispersion and polar components, respectively, of the SE of the test liquid ($\gamma_{l}$). Similarly, $\gamma_{s}^{d}$ and $\gamma_{s}^{p}$ are the dispersion and polar components, respectively, of the SE of the solid ($\gamma_{s}$).

The optical properties, such as transmission, of the films were investigated using a spectral measurement system (OtO Photonics, Spectra Smart, Collimage, Taipei, Taiwan). The hysteresis loop of CoFeSm films was measured via vibrating sample magnetometer (VSM, NanoMagnetics, Ulm, Germany). The mechanical properties such as hardness and Young’s modulus of the thin films were measured via the nanoindentation technique (KLA, iNano^®^, MTS, Minneapolis, MN, USA). The MTS Nano Indenter XP with a Berkovich tip and continuous stiffness measurement (CSM) techniques was used to measure the hardness and Young’s modulus. Before the indenter was gradually removed from the surface, the loading was reduced to 10% of the maximum load. Ten distinct measurements were taken via the indenter for each sample. The indentation depth did not exceed 30% of the films’ thickness to avoid the influence of the substrate effect on mechanical properties [22].

The hardness (*H*) and reduced elastic modulus ($E_{r}$) can be defined by the Equations (2) and (3) shown below [22]:

(2) $$H=\frac{P_{max}}{A_{p}}$$

(3) $$E_{r}=\frac{1}{2}S\sqrt{\pi /A_{p}\;}$$

where $P_{max}$ is maximum indentation load, $A_{p}$ is the projected contact area, and S is the contact stiffness.

The elastic modulus of thin films ($E_{f}$) was then calculated as the following equation, which is shown in Equation (4) below [22]:

(4) $$\;\;\;\;E_{f}=\left(1-\upsilon_{f}^{2}\right){\left(\frac{1}{E_{r}}-\frac{1-\upsilon_{i}^{2}}{E_{i}}\right)}^{-1}$$

Poisson’s ratio (υ) and the subscripts “i” and “f” are denoted as the parameters for indenter and measured films. For the diamond indenter tip, $E_{i}=1141$ GPa, $\upsilon_{i}$ = 0.07, and $\upsilon_{f}$ were taken and were equal to 0.25.

## 3. Results

**Figure 1:**
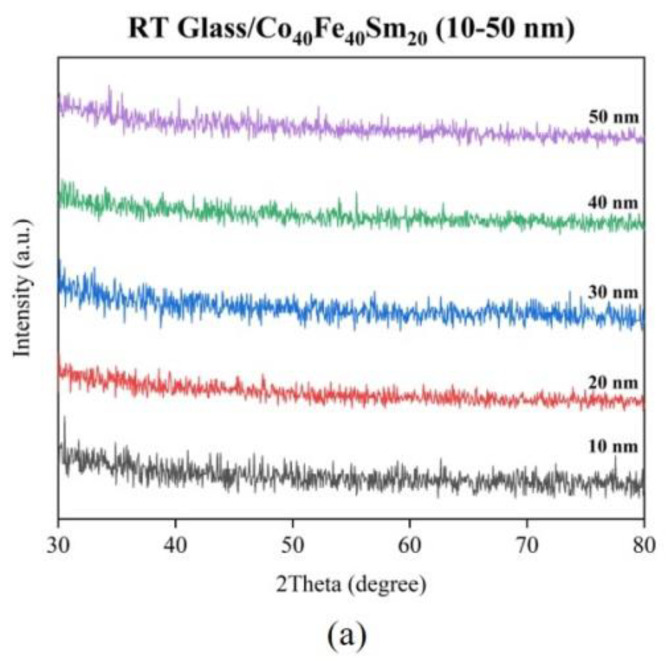

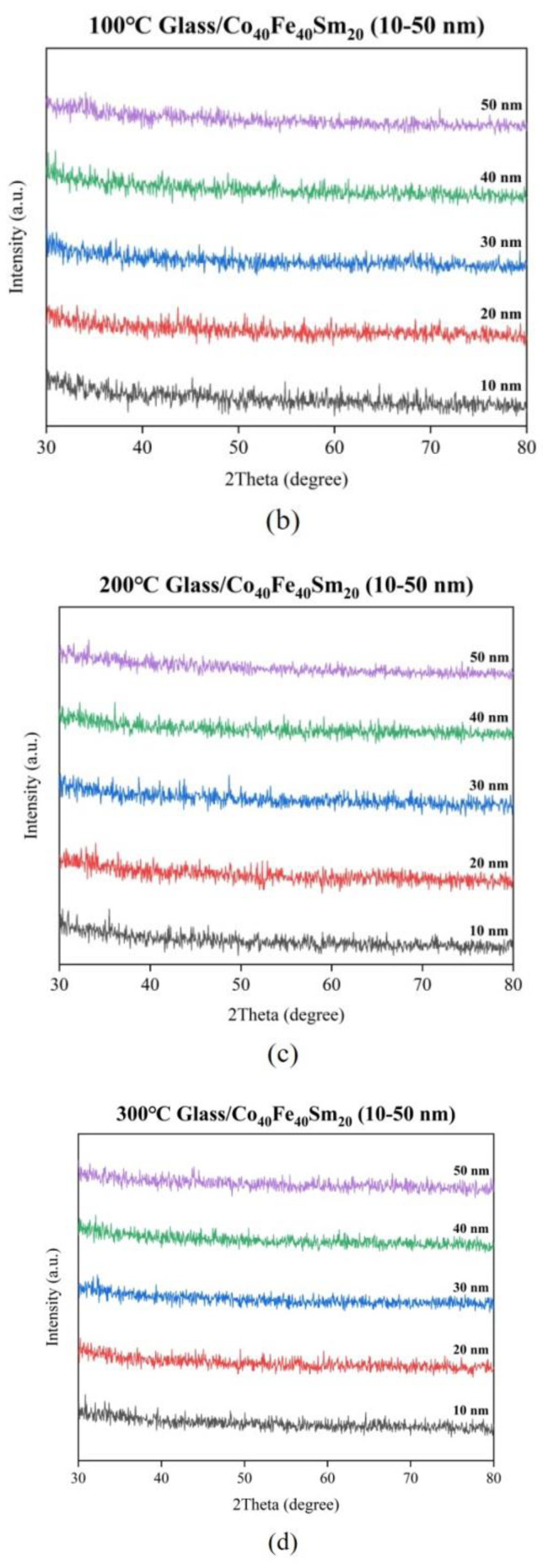
X-ray diffraction patterns of Co_40_Fe_40_Sm_20_ (10–50 nm) films annealed at different temperatures (**a**) RT, (**b**) 100 °C, (**c**) 200 °C, and (**d**) 300 °C.

Figure 1 represents the XRD patterns of (a) as-deposited and (b–d) annealed Co_40_Fe_40_Sm_20_ films. It can be observed from the XRD pattern that there are no obvious XRD peaks for any of the Co_40_Fe_40_Sm_20_ films, indicating that all the sample films are amorphous. The study shows that FeCoSm films exist in an amorphous state when the Sm content is higher than 13% [23]. This emphasizes that the crystal structure is difficult to grow on an amorphous substrate compared with that of a crystalline substrate. For this reason, the structure of all the Co_40_Fe_40_Sm_20_ films on the glass substrate was in an amorphous state [24]. The reference shows that the energy of sputtered atoms increases with increasing sputtering power and facilitates crystal structure. The highly energized electrons with increasing sputtering power will bombard the surface of the growing film at the substrate, providing thermal energy. This energy might act as an additional energy to promote crystallization growth [24,25]. Hence, the amorphous state of Co_40_Fe_40_Sm_20_ films may be due to the low sputtering power, 50 W, and insufficient thermal energy. Low sputtering power results in amorphous structure when it comes to the impact of deposition power on the structural characteristics of the deposited films.

**Figure 2:**
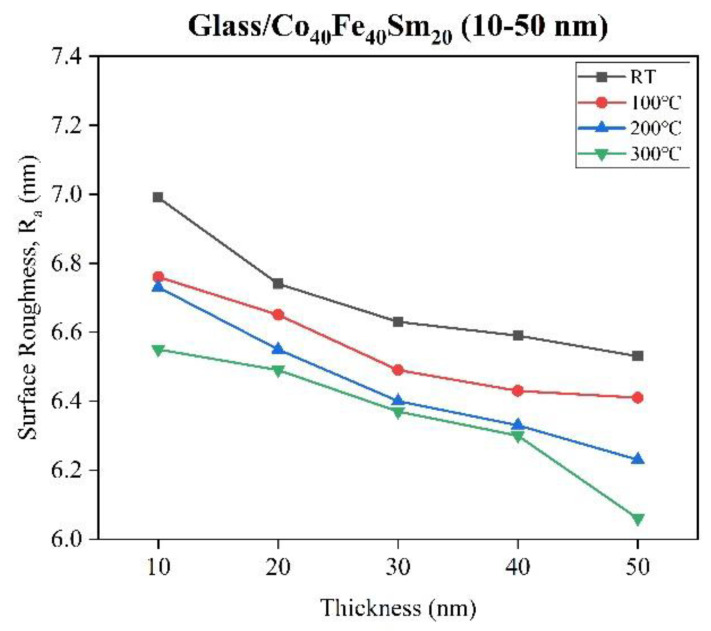
Surface roughness of as-deposited and annealed Co_40_Fe_40_Sm_20_ (10–50 nm) films.

**Figure 3:**
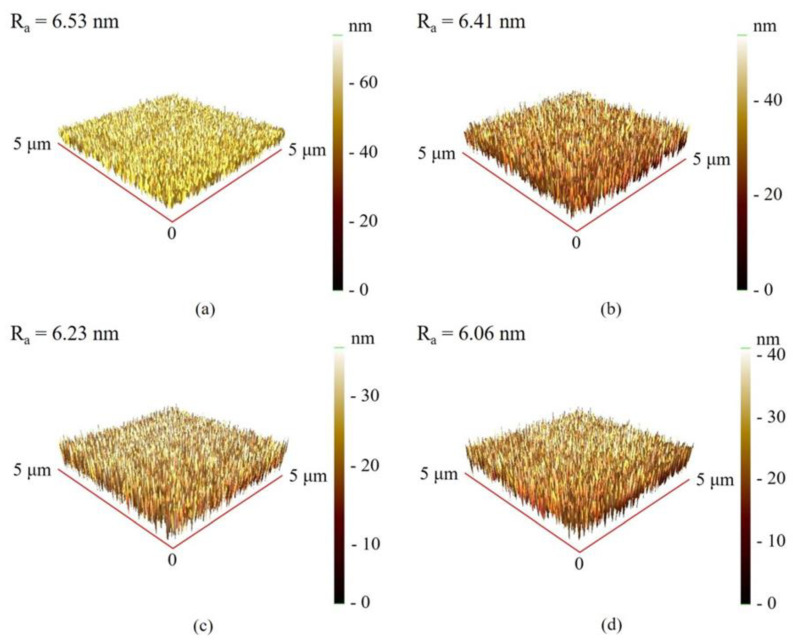
AFM images of 50 nm Co_40_Fe_40_Sm_20_ thin films annealed at different temperatures (**a**) RT, (**b**) 100 °C, (**c**) 200 °C, and (**d**) 300 °C.

Figure 2 shows the surface roughness of as-deposited and annealed Co_40_Fe_40_Sm_20_ films with different thicknesses obtained over a scanning area of 5 μm × 5 μm. AFM images of as-deposited and annealed 50 nm Co_40_Fe_40_Sm_20_ films are displayed in Figure 3. This finding shows that the R_a_ of Co_40_Fe_40_Sm_20_ films decreases with increased thickness of the film from 10 nm to 50 nm and annealing temperatures up to 300 °C. Thus, the 50 nm Co_40_Fe_40_Sm_20_ films annealed at 300 °C has a smoother surface. The R_a_ decreases with increased film thickness—a consequence of the minimization of compressive strain [26]. The surface smoothening results from the surface diffusion of volume or surface of adsorbed atoms. As the annealing temperature increases, the atoms become more energetic and migrate faster on the substrate surface, indicating that the surface mobility of atoms is increased. The increased mobility of atoms on the substrate encourages a more homogeneous surface and causes the surface roughness to decrease [27,28].

**Figure 4:**
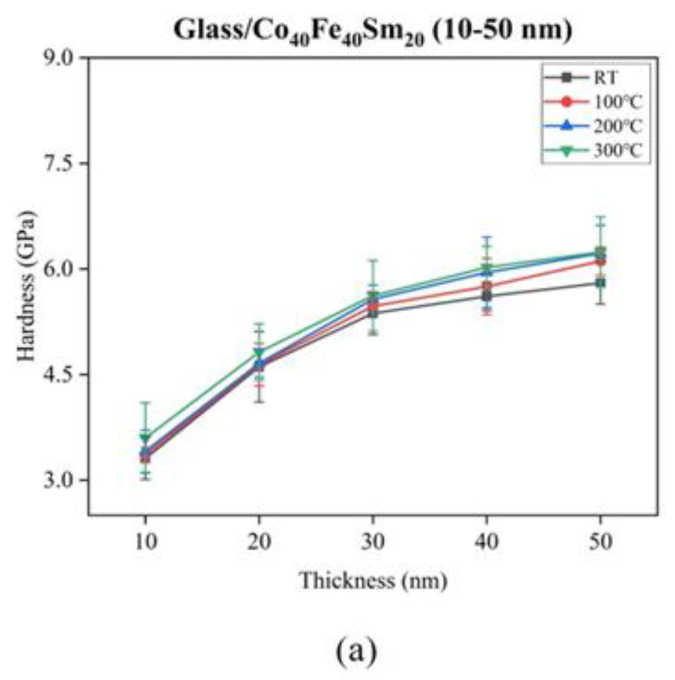

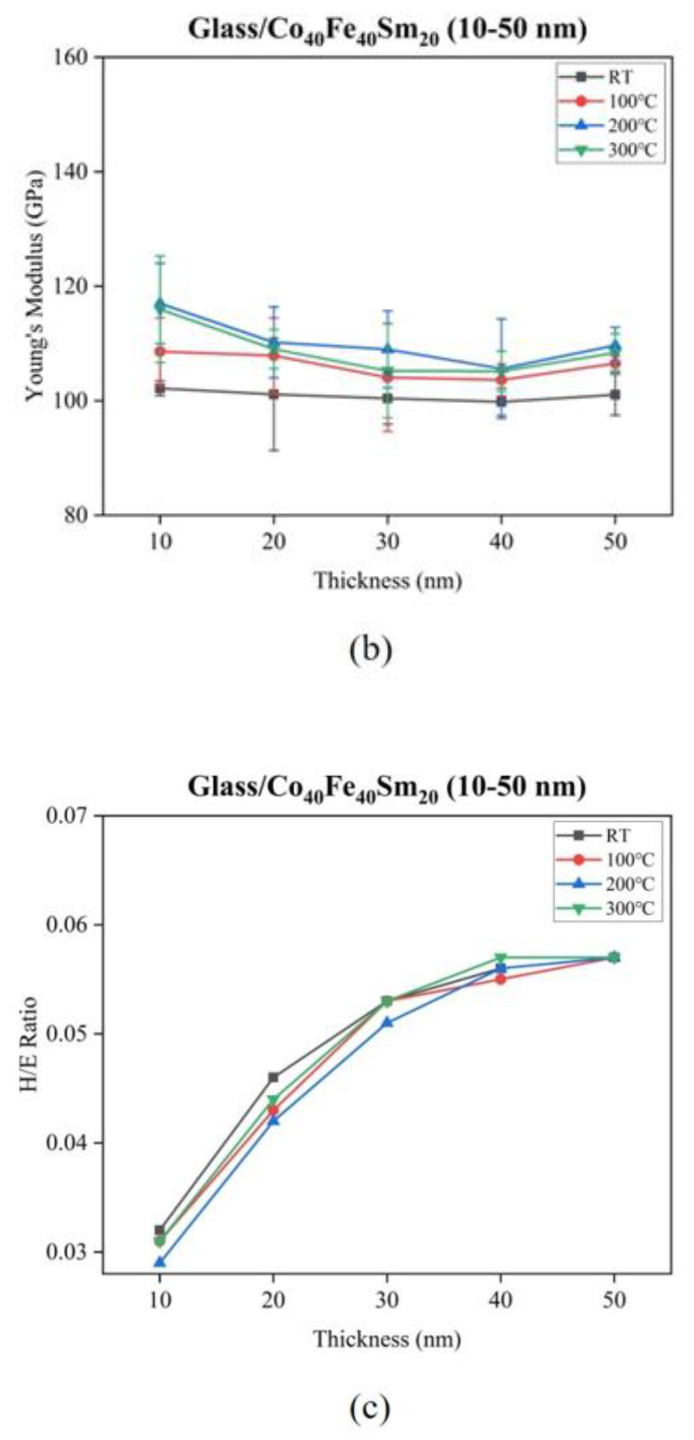
(**a**) Hardness, (**b**) Young’s modulus, and (**c**) H/E ratio of as-deposited and annealed Co_40_Fe_40_Sm_20_ (10–50 nm) films.

Nanoindentation measures mechanical characteristics, such as the hardness (H) and Young’s modulus (E) of Co_40_Fe_40_Sm_20_ films. Hardness measures a material’s resistance to localized plastic deformation. Young’s modulus may be considered a measure of stiffness, depending on the interatomic distances [29]. The hardness ratio over Young’s modulus (H/E ratio) in the films can be used to measure the ability of a material to resist plastic deformation in a contact event. Resistance to contact damage not only depends on the H but also the E value; contact damage can be avoided by a material with high H and low E [30]. It should be noted that the penetration depth of the indenter must be less than 30% of the thickness of the film; otherwise, the hardness of thin films would be affected by the hardness of the substrate [31]. Figure 4 shows the (a) hardness, (b) Young’s modulus, and (c) H/E ratio of as-deposited and annealed Co_40_Fe_40_Sm_20_ films with film thicknesses ranging from 10 nm to 50 nm, respectively. As shown in Figure 4a, the H values of Co_40_Fe_40_Sm_20_ films increase when film thickness increases from 10 nm to 50 nm and annealing temperatures increase from RT to 300 °C. This can be explained by the amorphous metals’ possessing nano-crystalization [32]. From Figure 4b, the E values of Co_40_Fe_40_Sm_20_ films are decreased from 10 nm to 40 nm and slightly increased at 50 nm. The E values of Co_40_Fe_40_Sm_20_ films are increased from RT to 200 °C and then decreased when the annealing temperature rises to 300 °C. It is well known that the E value is intrinsically determined by the interatomic distances and average atomic radius, as shown in Equation (5) below:

(5) $$E=\frac{1}{r_{0}}\left[\frac{1}{r}\left(\frac{dU}{dt}\right)\right]$$

where E and U are the elastic modulus and interatomic distances, respectively; $r_{0}$ and r are the average atomic distance and the bonding length between two atoms, respectively.

Based on Equation (5), a decrease in the E value can be attributed to increased atomic bonding length due to a less-dense atomic packing state [33]. As shown in Figure 4c, the H/E ratio of 40 nm Co_40_Fe_40_Sm_20_ films annealed at 300 °C reached a maximum value up to 0.057. The high ratio of the H/E parameter is an indication of high plastic deformation. Thus, these alloy films are expected to exhibit higher wear resistance characteristics [34]. Materials with a low elastic modulus will even out easily against a substrate and make notable contact. Therefore, they strongly adhere to the substrate. Hence, this demonstrates that Young’s modulus decreases and increases larger surface energy, indicating a better adhesion to the substrate [35,36]. The average error value of H/E ratio was ±0.001. The value of 0.057 ± 0.001 for the H/E ratio is compared to the higher ratio of CoFeNi films, which indicates that this H/E ratio is larger than that of CoFeNi films [37].

**Figure 5:**
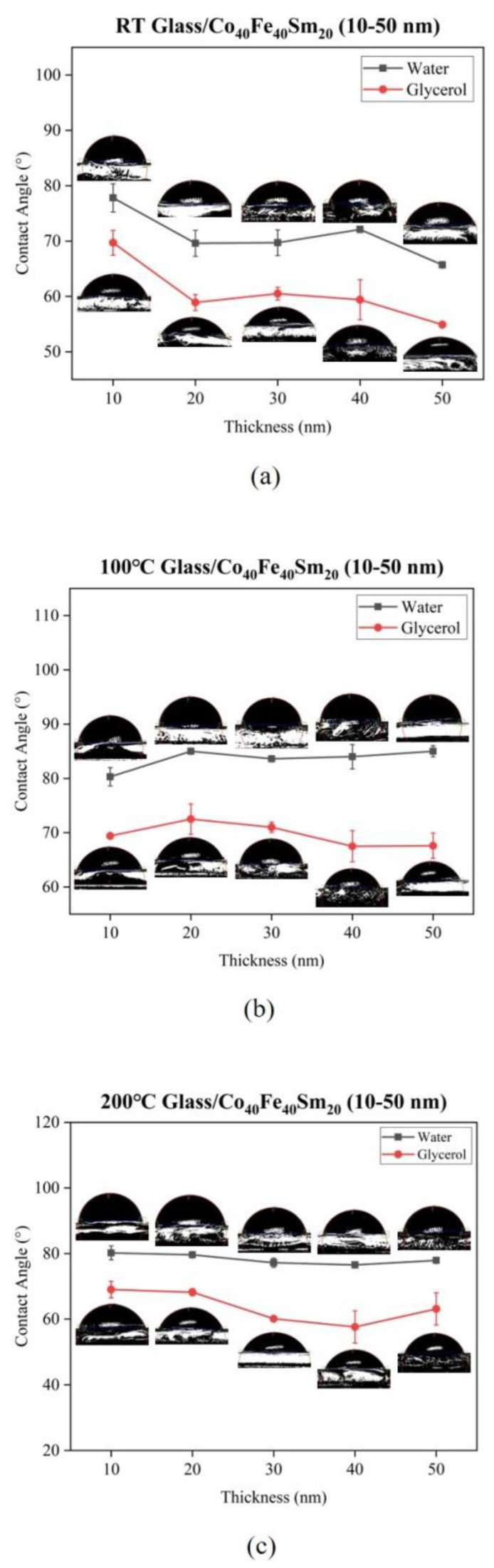

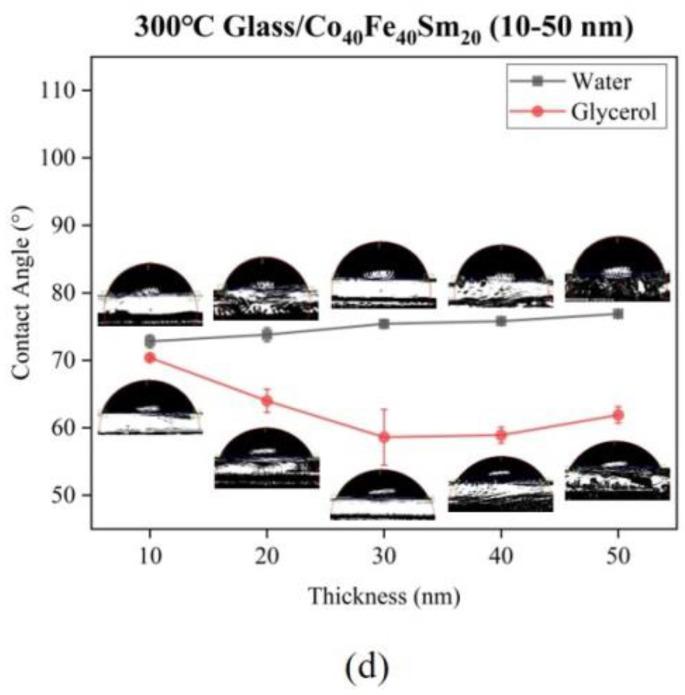
Contact angle of water and glycerol on Co_40_Fe_40_Sm_20_ (10–50 nm) films annealed at different temperatures (**a**) RT, (**b**) 100 °C, (**c**) 200 °C, and (**d**) 300 °C.

**Figure 6:**
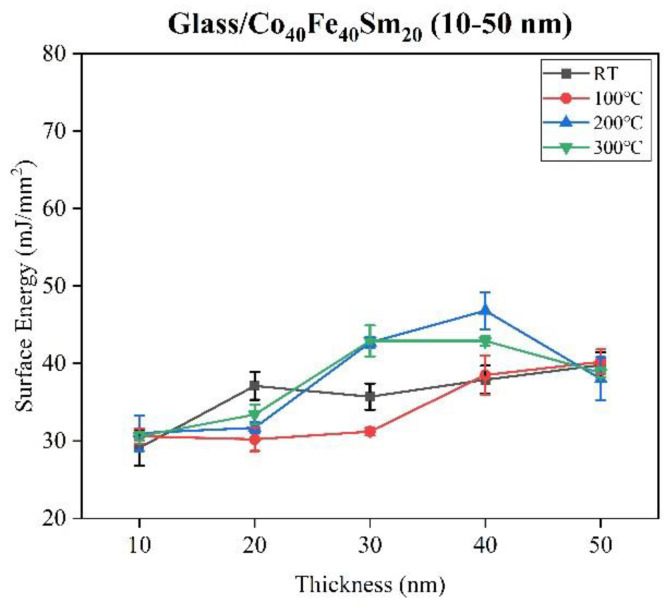
Surface energy of as-deposited and annealed Co_40_Fe_40_Sm_20_ (10–50 nm) films.

Figure 5 shows the contact angle of water and glycerol for as-deposited and annealed Co_40_Fe_40_Sm_20_ films with film thickness ranging from 10 nm to 50 nm. The contact of water and glycerol for all the Co_40_Fe_40_Sm_20_ films was determined to calculate the surface energy of Co_40_Fe_40_Sm_20_ films using the Owens–Wendt–Kaelble model, as shown in Equation (1). The contact angle of water for all the Co_40_Fe_40_Sm_20_ films ranged from 65.7° to 85.0°, with a contact angle of less than 90°, indicating the hydrophilic nature of Co_40_Fe_40_Sm_20_ films. Figure 6 represents the surface energy of as-deposited and annealed Co_40_Fe_40_Sm_20_ films. The 40 nm Co_40_Fe_40_Sm_20_ films annealed at 200 °C showed the highest surface energy values, namely 46.8 mJ/mm^2^. The larger the surface energy, the lower the surface tension of the liquid, and the attraction force between the liquid molecules and the atoms in the solid is stronger than the attraction force between the liquid molecules. Thus, higher surface energy means an easier wetting process and higher adhesion [21]. Hence, the 40 nm Co_40_Fe_40_Sm_20_ films annealed at 200 °C showed better adhesive properties than the others. However, in the 40 nm films annealed at 200 °C, the behavior is different with respect to other samples, which could reasonably possibly be due to changes in surface morphology [38]. Surface energy and adhesion are significant factors because Co_40_Fe_40_Sm_20_ film can be used as a buffer or seed layer, which can be combined with other layers to form a multilayered structure. The contact angle decreases due to significant liquid absorption when the surface energy is high.

**Figure 7:**
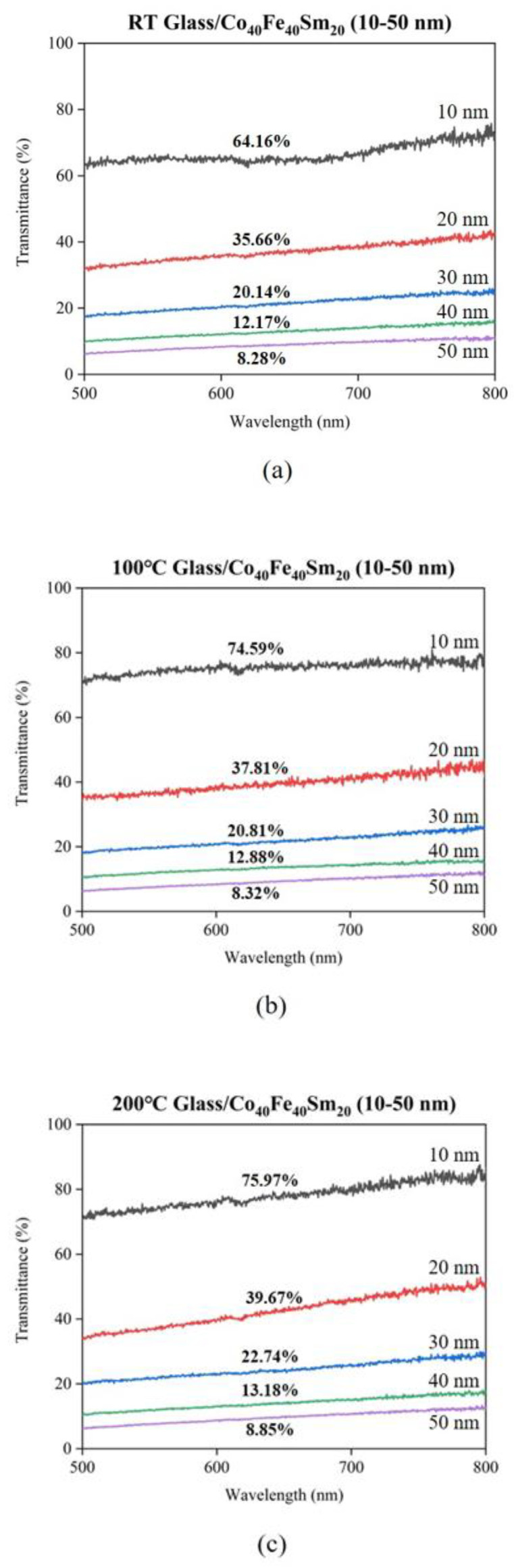

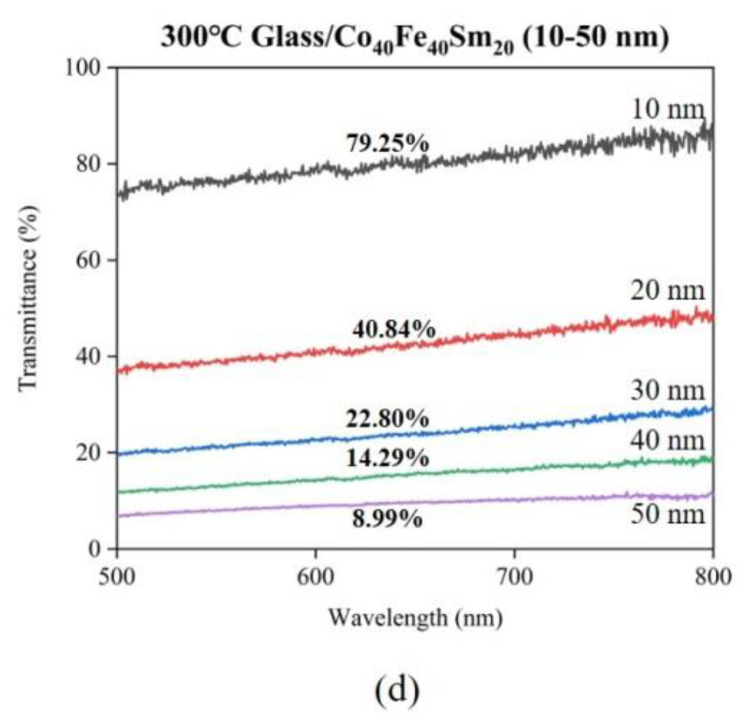
Transmittance of Co_40_Fe_40_Sm_20_ (10–50 nm) films annealed at different temperatures (**a**) RT, (**b**) 100 °C, (**c**) 200 °C, and (**d**) 300 °C.

**Figure 8:**
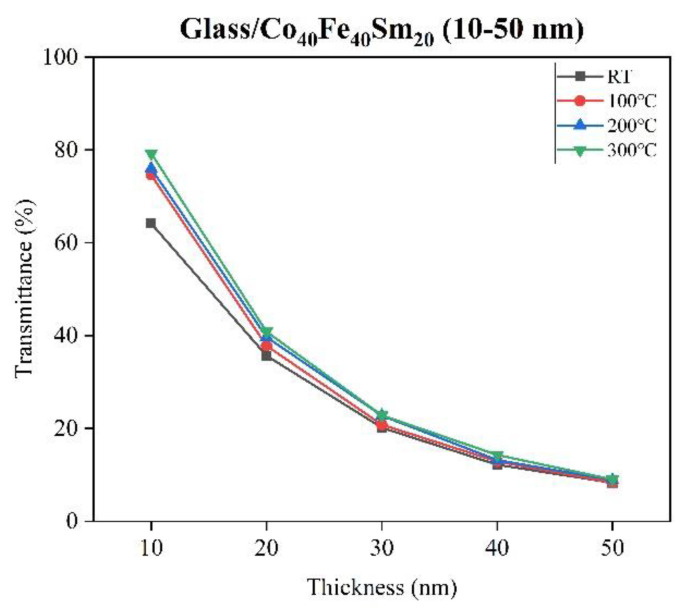
Transmittance of as-deposited and annealed Co_40_Fe_40_Sm_20_ (10–50) films with 600 nm of wavelength.

**Figure 9:**
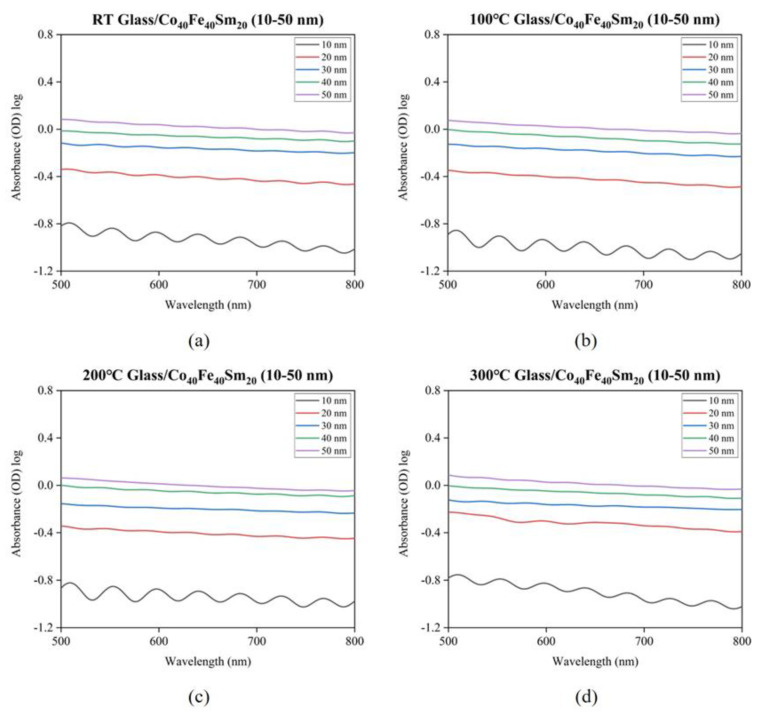
Absorbance of Co_40_Fe_40_Sm_20_ (10–50 nm) films annealed at different temperatures (**a**) RT, (**b**) 100 °C, (**c**) 200 °C, and (**d**) 300 °C.

The transmittance and absorbance of Co_40_Fe_40_Sm_20_ films were studied via a spectral measurement system as a function of wavelength in a range of 500 to 800 nm, as shown in Figure 7, Figure 8 and Figure 9, respectively. In contrast, Figure 8 displays the transmittance of Co_40_Fe_40_Sm_20_ films at wavelengths of 600 nm. Figure 9 reveals the absorbance of Co_40_Fe_40_Sm_20_ films. It can be observed that the transmittance of Co_40_Fe_40_Sm_20_ films decreased with increased film thickness from 10 nm to 50 nm, suggesting greater film thickness may inhibit the transfer of photo signals through the films and cause high transmittance and low absorbance [39]. The interference bands in optical absorption are not observed in particular in the thicker samples due to the light-scattering effects related to the surface roughness [40]. A smoother surface reduces light scattering and reflection, implying greater light absorption and improved optical performance, which is consistent with the ZnO optical result [41]. The transmittance of Co_40_Fe_40_Sm_20_ films increased with increasing annealing temperatures from RT to 300 °C. This is because the scattering behavior of the films was improved in rougher films. This may cause a greater number of photons to be scattered on the lattice surface and decrease the transmittance of Co_40_Fe_40_Sm_20_ films [42]. Hence, this increased transmittance was due to the smoother surface of Co_40_Fe_40_Sm_20_ films consisting of small and fine particles. The surface roughness (R_a_), hardness, Young’s modulus, H/E ratio, surface energy (SE), and transmittance of Co_40_Fe_40_Sm_20_ films annealed at different temperatures (T_a_) are summarized in Table 2. Surface roughness is an important factor that affects wetting behavior, which can improve the surface’s hydrophobicity. The results indicate that surface roughness is decreased with increased thicknesses and annealing temperatures. Results showed that as the surface roughness decreased, both the contact angle decreased and the surface energy increased [43,44].

**Figure 10:**
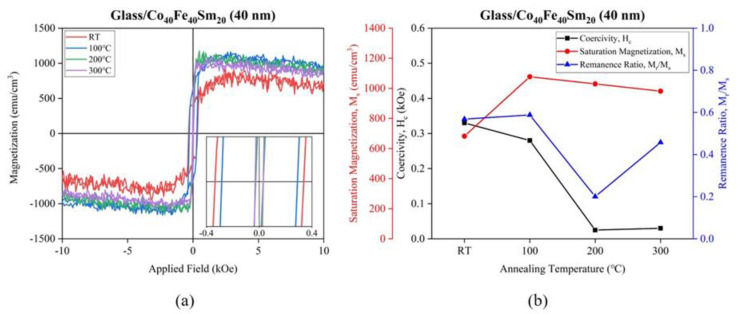
(**a**) In-plane hysteresis loop and (**b**) variations of coercivity, saturation magnetization, and remanence ratio for in-plane magnetization of deposited and annealed Co_40_Fe_40_Sm_20_ (40 nm) films.

The Co_40_Fe_40_Sm_20_ (40 nm) films annealed at 200 °C showed higher surface energy and reached a relatively high H/E ratio; therefore, the in-plane hysteresis loop of 40 nm Co_40_Fe_40_Sm_20_ films were conducted via VSM. Figure 10a shows the in-plane hysteresis loop of 40 nm Co_40_Fe_40_Sm_20_ films, and Figure 10b shows the plot of the coercivity and saturation magnetization for in-plane magnetized 40 nm Co_40_Fe_40_Sm_20_ films annealed at various temperatures. Table 3 listed the H_c_, M_s_, and remanence ratio (M_r_/M_s_) of Co_40_Fe_40_Sm_20_ (40 nm) thin films annealed at different temperatures. The H_c_ values decreased from 0.330 kOe to 0.025 kOe when the annealing temperatures increased from RT to 200 °C and slightly increased to 0.030 kOe when annealed at 300 °C. The Co_40_Fe_40_Sm_20_ films with finer grain exhibited lower coercivity and followed the power law H_c_ ∝ D^6^ [45]. Thus, the H_c_ values decreased with increasing annealing temperatures, possibly because of the decrease in crystal size in 40 nm Co_40_Fe_40_Sm_20_ films with increased annealing temperatures from RT to 300 °C. The 40 nm Co_40_Fe_40_Sm_20_ films that were annealed at 200 °C have the lowest H_c_ and relatively high M_s_, resulting in better soft magnetic properties, and are suitable for spintronics, micro-actuators, magnetic memories, and storage devices. Additionally, the 40 nm Co_40_Fe_40_Sm_20_ films that were annealed at 200 °C showed the lowest M_r_/M_s_ ratio, resulting in the magnetic moment easily reducing magnetization to zero when the external magnetic field was removed. Moreover, Table 4 shows the comparison between the coercivity (H_c_) of Co_40_Fe_40_Sm_20_ (40 nm) film with Sm_37.7_Co_62.3_ and Co_40_Fe_40_Y_20_ films.

## 4. Conclusions

Co_40_Fe_40_Sm_20_ films were deposited via DC magnetron sputtering techniques with different film thicknesses and then annealed at different temperatures, up to 300 °C, to study their microstructural, mechanical, and optical properties and wettability. AFM showed that the surface roughness of Co_40_Fe_40_Sm_20_ films decreased with increasing film thickness and annealing temperatures. The nanoindentation technique showed that the hardness of Co_40_Fe_40_Sm_20_ films increased with increasing film thickness and annealing temperature. A maximum H/E ratio was reached in 40 nm Co_40_Fe_40_Sm_20_ films annealed at 200 °C, which are expected to have better wear resistance characteristics. Contact angle measurement demonstrated hydrophilic characteristics in all the Co_40_Fe_40_Sm_20_ films. The 40 nm Co_40_Fe_40_Sm_20_ films annealed at 200 °C obtained higher surface energy, resulting in better adhesion to the substrate. Furthermore, the smoother surface morphology showed higher transmittance values, which gave the Co_40_Fe_40_Sm_20_ films more transparency.

## Figures and Tables

**Table 1:** Significant properties for CoFeV, CoFeW, CoFeYb, CoFeY, and CoFeSm films.

Materials	Maximum χ_ac_ (a.u.)	Surface Energy (mJ/mm^2^)	Transmittance (%)
Glass/Co_40_Fe_40_V_20_ [15,16] 10–100 nm at RT	0.02–0.04	27.8–45.4	x
Glass/Co_32_Fe_30_W_38_ [17] 10–50 nm at RT and annealed conditions	0.02–0.52	22.3–28.4	x
Glass/Co_40_Fe_40_Yb_20_ [18] 10–50 nm at RT and annealed conditions	0.04–0.35	28.6–34.5	22.3–80.5
Si(100)/Co_40_Fe_40_Y_20_ [19] 10–50 nm at RT and annealed conditions	0.03–0.16	22.7–31.1	x
Glass/Co_40_Fe_40_Y_20_ [20] 10–50 nm at RT and annealed conditions	0.04–0.20	23.3–28.7	20.1–81.7
Glass/Co_40_Fe_40_Sm_20_ 10–50 nm at RT and annealed conditions (Current research)	x	29.1–46.8	8.2–79.2

**Table 2:** Surface roughness (R_a_), hardness (H), Young’s modulus (E), H/E ratio, surface energy (SE), and transmittance of Co_40_Fe_40_Sm_20_ (10–50 nm) films annealed at different temperatures (T_a_).

T_a_ (°C)	Thickness (nm)	R_a_ (nm)	H (GPa)	E (GPa)	H/E ratio	SE (mJ/mm^2^)	Transmittance (%)
RT	10	6.99	3.31	102.17	0.032	29.1	64.16
	20	6.74	4.61	101.12	0.046	37.1	35.66
	30	6.63	5.37	100.42	0.053	35.7	20.14
	40	6.59	5.61	99.80	0.056	37.9	12.17
	50	6.53	5.80	101.05	0.057	39.8	8.28
100	10	6.76	3.36	108.58	0.031	30.6	74.59
	20	6.65	4.64	107.88	0.043	30.2	37.81
	30	6.49	5.47	104.07	0.053	31.2	20.81
	40	6.43	5.75	103.63	0.055	38.5	12.88
	50	6.41	6.11	106.50	0.057	40.2	8.32
200	10	6.73	3.41	117.00	0.029	31.0	75.97
	20	6.55	4.66	110.20	0.042	31.7	39.67
	30	6.40	5.57	108.97	0.051	42.7	22.74
	40	6.33	5.95	105.60	0.056	46.8	13.18
	50	6.23	6.22	109.68	0.057	38.0	8.85
300	10	6.55	3.60	115.98	0.031	30.6	79.25
	20	6.49	4.82	109.02	0.044	33.4	40.84
	30	6.37	5.62	105.22	0.053	42.9	22.80
	40	6.30	6.02	105.16	0.057	42.9	14.29
	50	6.06	6.24	108.39	0.057	38.8	8.99

**Table 3:** Coercivity (H_c_), saturation magnetization (M_s_), and remanence ratio (M_r_/M_s_) of Co_40_Fe_40_Sm_20_ (40 nm) films with different annealing temperature (T_a_).

T_a_ (°C)	H_c_ (kOe)	M_s_ (emu/cm^3^)	M_r_/M_s_
RT	0.330	682.13	0.57
100	0.280	1076.93	0.59
200	0.025	1029.24	0.20
300	0.030	981.13	0.46

**Table 4:** Comparison between the coercivity (H_c_) and saturation magnetization (M_s_) of Co_40_Fe_40_Sm_20_ film with Sm_37.7_Co_62.3_ and Co_40_Fe_40_Y_20_ films.

Materials	Substrate Types	Annealing Temperatures (°C)	H_c_ (kOe)	M_s_ (emu/cm^3^)
Co_40_Fe_40_Sm_20_ (40 nm) films	Glass	RT	0.330	682.13
		100	0.280	1076.93
		200	0.025	1029.24
		300	0.030	981.13
Sm_37.7_Co_62.3_ (70 nm) films [9]	Glass	RT	37.938	150
Co_40_Fe_40_Y_20_ (50 nm) films [20]	Glass	RT	-	482
		300		610

## Data Availability

Not applicable.
